# Bibliometric network mapping of farm accounting studies using a comprehensive dataset

**DOI:** 10.1016/j.dib.2024.110288

**Published:** 2024-03-01

**Authors:** Mustafa Kıllı, İlker Kefe

**Affiliations:** International Trade and Logistic Department, Faculty of Economics and Administrative Sciences, Osmaniye Korkut Ata University, Osmaniye, Turkey

**Keywords:** Farm accounting research, Biological assets, Fair value, Bibliometric analysis, VOSviewer

## Abstract

This data article aims to analyze the intellectual structure of farm accounting studies by examining bibliometric features. A dataset comprising 190 documents from the ISI Database within the farm accounting field was utilized. It delved into various aspects including the yearly publication and citation count concerning agricultural accounting, predominant research areas, keyword co-occurrence, bibliographic coupling among sources and documents, as well as co-citation patterns of referenced materials. Bibliometric network mapping techniques were employed for the analysis of the data. The analysis was conducted using VOSviewer, a scientific mapping analysis tool. The findings indicated a notable uptrend in publication and citation rates of agricultural accounting studies over the past decade. A significant portion of the dataset centered around agriculture and business economics. Key terms like ``biological assets,'' ``IAS 41,'' and ``fair value'' emerged as prominently used. The journal ``Custos e Agronegócio Online'' showed significant influence in terms of bibliographic coupling among sources.

Specifications Table

**Table:** 

Subject	*Social Sciences, Accounting*
Specific subject area	*Bibliometric data sourced from ISI database, pertaining to academic literature focused exclusively on farm accounting.* *Bibliometrics, Farm Accounting*
Data format	*Raw, Analysed*
Type of data	*Text* *Figure* *Table*
Data collection	*A data set from the ISI database is used to examine farm accounting studies. While determining the database in the research, criteria such as ease of access to the database, impact factors of the documents indexed in the database and the potential to direct the field, and the possibility of downloading data in the file type suitable for the program used for bibliometric analysis were taken into consideration. As the data source, ISI database is preferred because it provides these conditions. Data from farm accounting studies are analyzed by visual mapping method using the VOSviewer program.* *Farm accounting studies in the ISI database have been researched on a subject-based. In the filtering process of the study, the keywords ``farm accounting'', ``agricultural accounting'', ``IAS 41'' were entered in the subject part (title, summary and keywords). Since the research data were obtained in May 2023, studies published in 2023 were not included in the scope of the research. The research sample includes 190 documents published as of the end of 2022.*
Data source location	*The bibliometric analysis method is employed using the ISI (Web of Science) database, which contains scientific journals, books, and conference proceedings and is well-established and widely recognized.*
Data accessibility	Repository name: *Mendeley Data* Data identification number: *Bibliometric Network Mapping of Farm Accounting Studies Using a Comprehensive Dataset* Direct URL to data: https://data.mendeley.com/datasets/b6ycptcycn/1

## 1 Value of the Data

• This data reveals the research trends in farm accounting studies over the years.
• This data identifies the authors, countries, and institutions that have made the most significant contributions to farm accounting research.
• This data offers insights into the distribution of farm accounting research based on document type, index, and research areas.
• This data identifies the most frequently used keywords in farm accounting research.
• This data reveals the subjects that have been studied the most in the field of farm accounting research.
• This data thoroughly examines and describes the intellectual structure in the field of farm accounting research.
• The data set consisting of articles in scientific journals can be expanded with books or chapters while maintaining the same structure. Contributions in other languages or from journals not indexed in the ISI database may also be included.
• Focusing on farm accounting studies, the procedure for compiling data presented in this article can be used as a guide for similar studies conducted in different topics or disciplines.

## 2 Data Description

In this article, a data set from the ISI database is used to examine farm accounting studies. While determining the database, criteria such as ease of access to the database, impact factors of the documents indexed in the database, potential to guide the field, and the possibility of downloading data in a file type suitable for the program used are considered. ISI database was preferred as the data source because it meets these conditions for bibliometric analysis. The research aimed to determine the current state, development, and orientation of the field and establish its structure. Several aspects of farm accounting studies were analyzed, including the number of publications and citations over the years, the authors, institutions, and countries contributing the most to the field, publication types, indexes, and research areas, keyword co-occurrence, bibliographic coupling of sources and documents, and co-citation analysis of references. This data provides the list of articles published from 1983 to 2023 related to farm accounting. While determining the database in the research, criteria such as ease of access to the database, impact factors of the documents indexed in the database and the potential to direct the field, and the possibility of downloading data in the file type suitable for the program used for bibliometric analysis were taken into consideration. As the data source, ISI database is preferred because it provides these conditions.

**Fig. 1:**
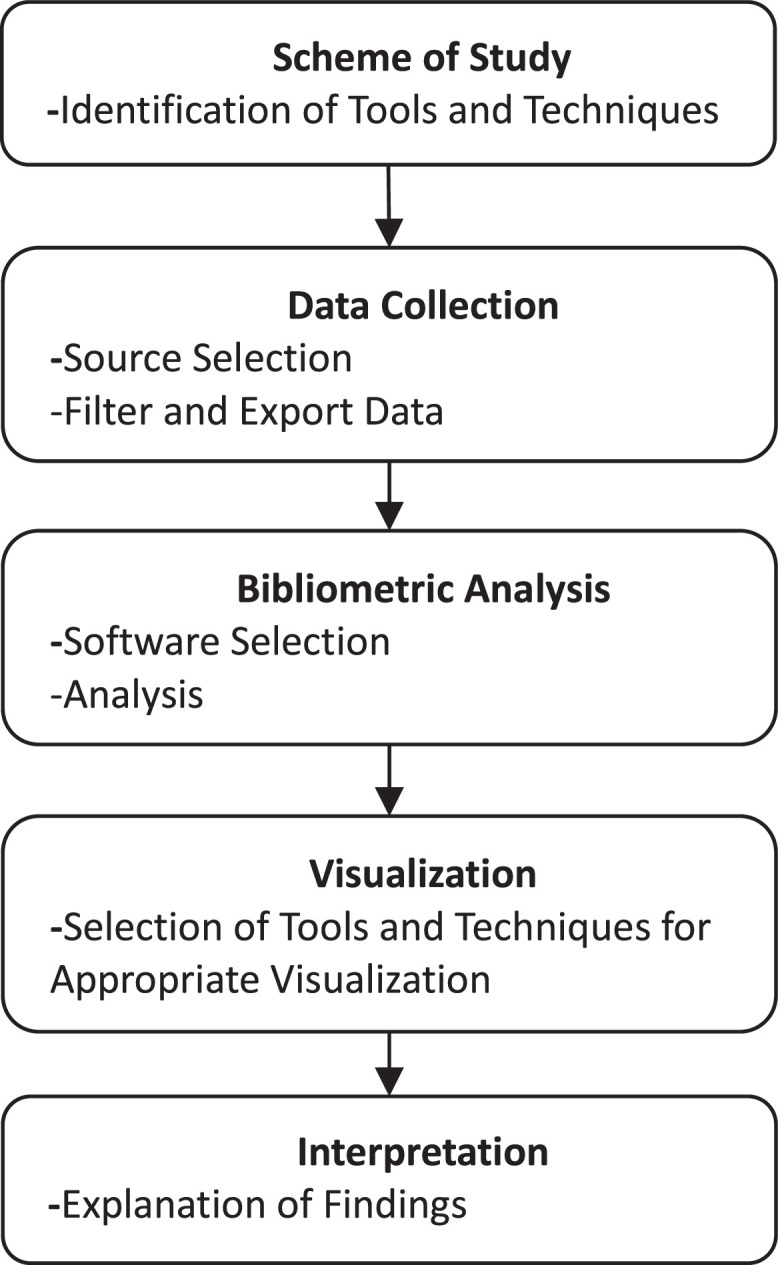
The proposed method for analysis.

The dataset contains the publications related to farm accounting on social sciences. This data is composed of two categories, including raw and analyzed. The raw file is available at https://data.mendeley.com/datasets/b6ycptcycn/1. The additional research dataset file for farm accounting studies on the repository includes the following data: author(s) names, publication title, publication type (Article or Proceedings Paper), keywords, abstract, year of publication and, index type. Farm accounting studies in the ISI database have been researched on a subject-based. In the filtering process of the study, the keywords ``farm accounting'', ``agricultural accounting'', ``IAS 41'' were entered in the subject part (title, summary, and keywords). The research sample includes 190 documents published as of the end of 2022. The analysis part is composed of figures and tables. Fig. 1 represents the steps to complete the process of bibliometric analysis of farm accounting literature.

The study follows a systematic approach, commencing with the identification of tools and techniques essential for effective research. Bibliometric analysis was preferred as the method of this study because it provides the opportunity to examine scientific studies in a research field in terms of features such as author, institution and country of the author, co-authorship relationship and citation links. The data collection process involves meticulous source selection, followed by the careful filtering and exportation of data, including study titles, keywords, and abstracts. Subsequently, the bibliometric analysis is conducted using the VOSviewer program, allowing for a thorough analytical examination. The next phase involves selecting suitable tools and techniques for clear data visualization. Finally, the interpretation phase unfolds, providing a comprehensive explanation of the results obtained throughout the entire process.

**Fig. 2:**
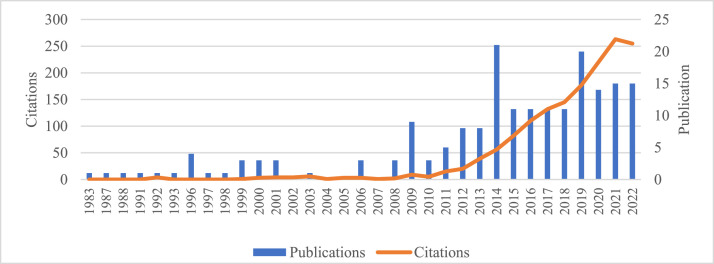
Publications and citations trends by years.

**Fig. 3:**
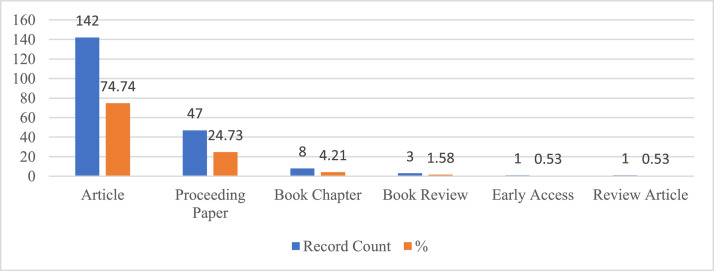
Distribution of publications by document type.

**Fig. 4:**
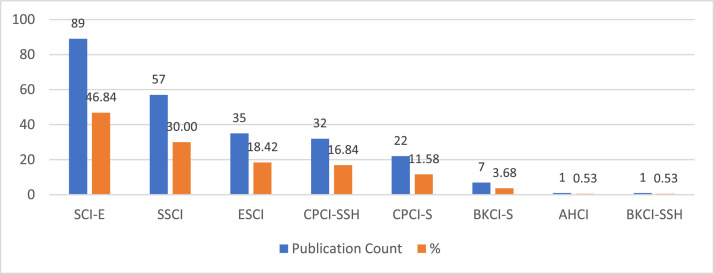
Distribution of publications by index.

**Fig. 5:**
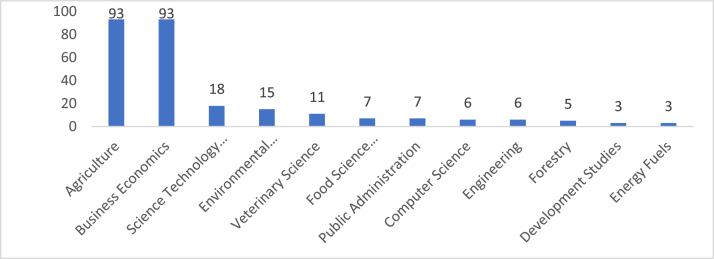
Publications numbers by research areas.

**Fig. 6:**
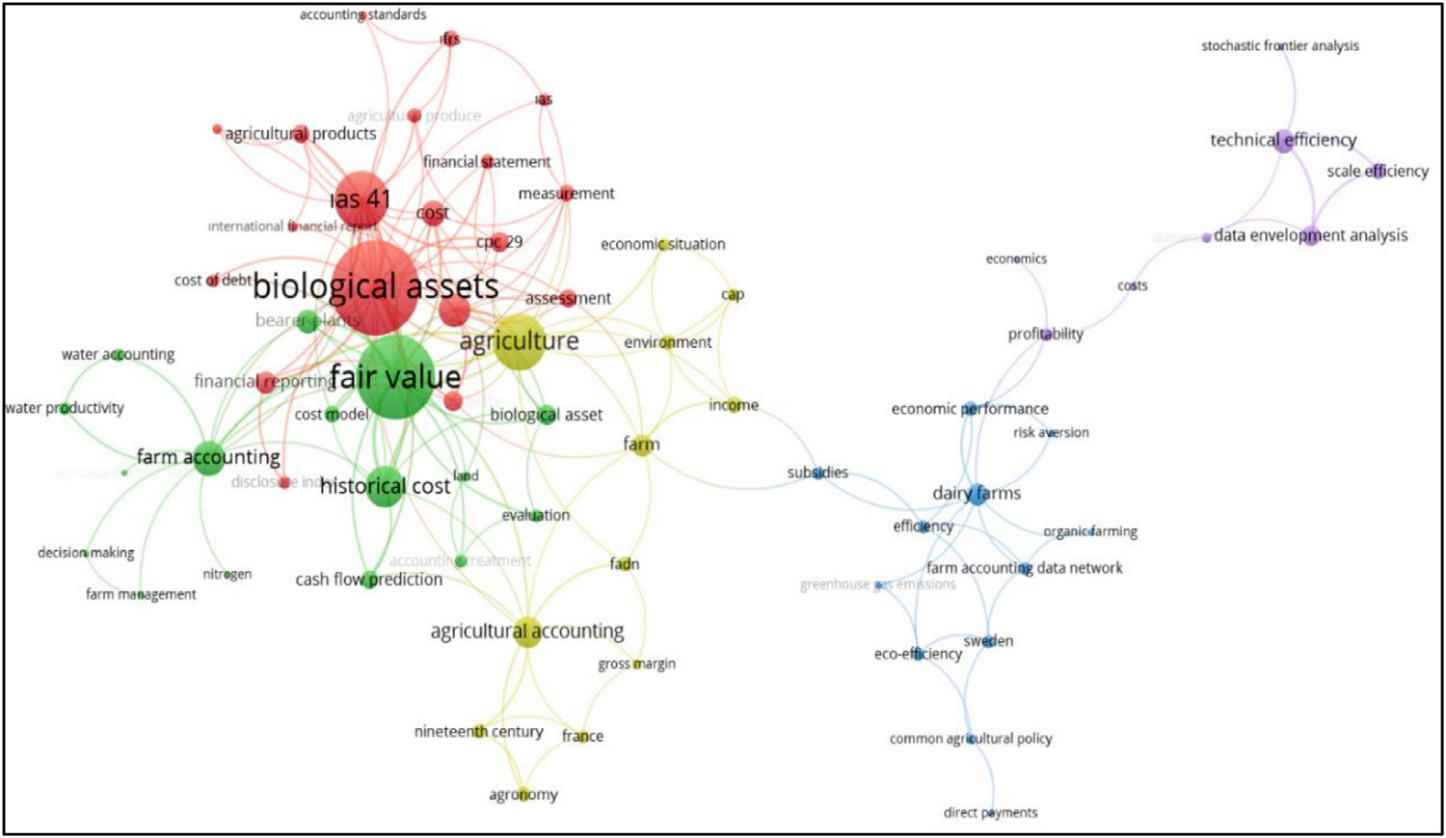
Network visualization of co-occurrence of keywords.

**Fig. 7:**
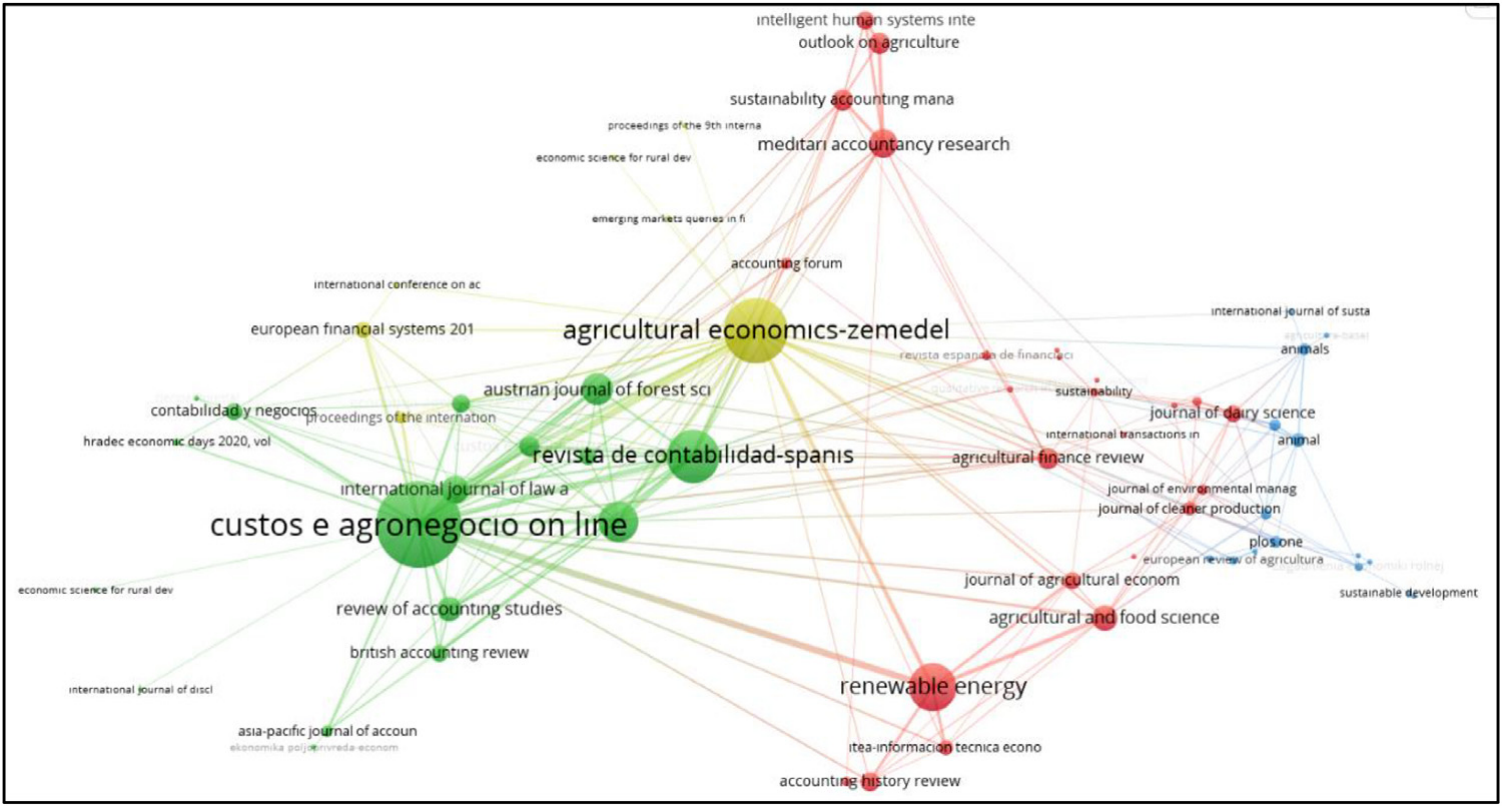
Network visualization of bibliographic coupling of sources.

**Fig. 8:**
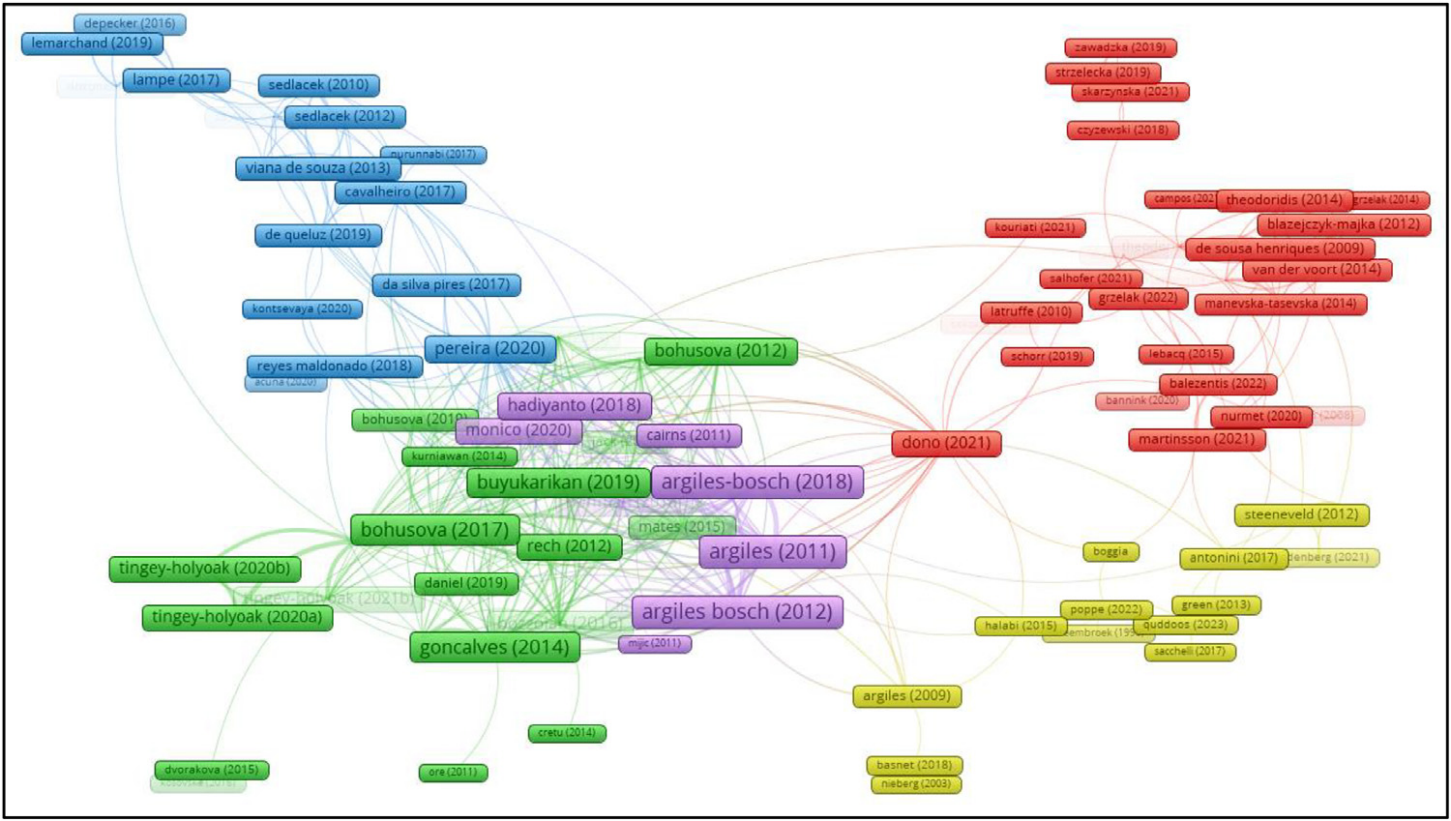
Network visualization of bibliographic coupling of documents.

**Fig. 9:**
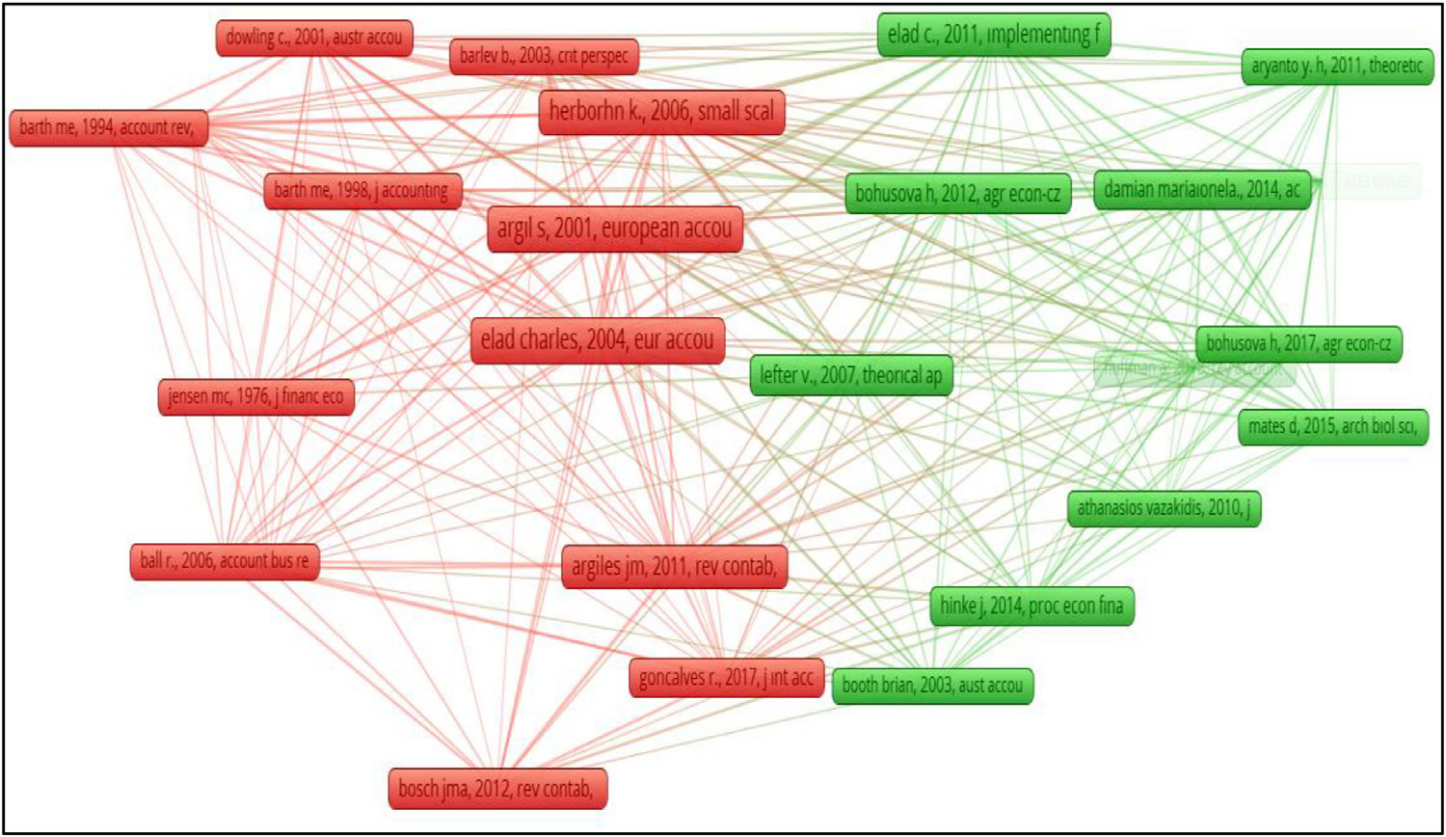
Network visualization of co-citation analysis of cited references.

Fig. 2 provides the publications and citation trends by year between 1983 and 2022. Fig. 3 provides the distribution of publications by document type, including articles, proceeding papers, book chapters, book reviews, and review articles. Fig. 4 provides the list of most cited articles, including the count of citations and count of citations per year. Fig. 5 provides the distribution of publications by index, including the Science Citation Index Expanded (SCI-EXPANDED), Social Sciences Citation Index (SSCI), Emerging Sources Citation Index (ESCI), Conference Proceedings Citation Index (CPCI-S, CPCI-SSH), Book Citation Index (BKCI-S, BKCI-SSH), and Arts and Humanities Citation Index (AHCI). Fig. 6 provides the network visualization of co-occurrence of keywords. Fig. 7 provides the network visualization of bibliographic coupling of sources. Fig. 8 provides the network visualization of bibliographic coupling of documents. Fig. 9 provides the network visualization of co-citation analysis of cited references. Table 1 provides the documents with the highest total links strength in bibliographic coupling. Table 2 provides the references with the highest total links strength in co-citation analysis.

**Table 1:** Documents with the highest total links strength in bibliographic coupling.

Rank	Documents	Citation	TLS
1	Argilés et al. (2011)	30	121
2	Argilés-Bosch et al. [1]	22	110
3	Argilés-Bosch et al. [2]	5	109
4	Goncalves & Lopes [3]	14	86
5	Bohusova & Svoboda (2017)	6	85
6	Tingey-Holyoak et al. (2021)	4	68
7	Buyukarıkan (2019)	2	67
8	Souza et al. (2013)	2	65
9	Bozzolan et al. (2016)	7	60
10	Hadiyanto et al. (2018)	4	56

**Table 2:** References with the highest total links strength in co-citation analysis.

Rank	References	Citation	TLS
1	Argilés & Slof [4]	17	92
2	Elad [5]	18	88
3	Herbohn & Herbohn (2006)	14	86
4	Argilés et al. (2011)	12	72
5	Elad & Herbohn [6]	12	63
6	Bohusova et al. [7]	9	58
7	Damian et al. [8]	7	51
8	Lefter & Roman (2007)	10	51
9	Dowling & Godfrey (2001)	6	48
10	Barlev & Haddad (2003)	5	45

## 3 Experimental Design, Materials and Methods

In this article, the data were analyzed by visual mapping method using VOSviewer program, which is a software developed for scientific mapping analysis. Research data, scientific network map was created by analyzing in the context of the co-occurrence status of the keywords, bibliographic coupling and co-citation analysis. The number of publications and citations of the farm accounting studies included in the research over the years shows in Fig. 1.

The number of farm accounting studies has shown a consistent increase over the past decade. In particular, 2014 and 2019 stand out with 21 and 20 publications, respectively, making them the years with the highest number of publications. Additionally, there has been a notable rise in the number of citations in correlation with the increase in publications over the same period. The year 2021 stands out as the most cited year, with a total of 63 citations.

Fig. 2 shows the distribution of the publications examined within the scope of the research by document types.

When the studies are examined by document type, it is seen that 74.74% (142) articles, 24.73% (47) papers, 4.21% (8) book chapters, 1.58% (3) book reviews, 0.53% (1) early access, 0.53% (1) review articles.

Fig. 3 presents distribution of the farm accounting studies by indexes in ISI Database. As shown in Fig. 3, 46.84% (89) of the published studies are indexed in the Science Citation Index Expanded (SCI-EXPANDED), 30% (57) are indexed in the Social Sciences Citation Index (SSCI), 18.42% are indexed Emerging Sources Citation Index (ESCI), 16.84% are indexed Conference Proceedings Citation Index–Social Science & Humanities (CPCI-SSH).

Fig. 4 displays the research areas of the farm accounting studies within the scope of the research.

When examining the research areas of farm accounting studies, it is observed that the majority of research is conducted in the fields of Agriculture (93) and Business Economics (93). Keyword co-occurrence analysis assumes that keywords that co-occur frequently in the same reviewed documents are related [9]. The analysis determines the strength and clusters of keywords in the network according to their co-occurrence. Items of the same color in the network represent clusters, and the size of the circles indicates the frequency of the terms. The closer the circles are in the same cluster, the more often the keywords are used together.

When examining the co-occurrence of keywords, a threshold of 2 was set as the minimum number of occurrences for a keyword to be included. Out of 502 keywords, 72 keywords met this criterion. The largest set of connected keywords consists of 67, as some of the 72 keywords in the network are not connected to each other. The co-occurrence analysis resulted in 5 clusters and a total of 168 links with a link strength of 270. The resulting network visualization is shown in Fig. 5.

The focused themes with reference to the keywords in the clusters are explained:

Cluster 1 (Red): There are 19 keywords in this cluster. ``Biological assets'' is the strongest keyword in this cluster, with 24 links and 77 total link strengths. ``IAS 41'' ranks second among the strongest keywords in this cluster with 16 links and 34 total link strength, and ``Accounting'' is third with 12 links and 16 total link strengths. This is followed by the keywords ``Cost'' with 6 links and 11 total link strength and ``Financial Reporting'' with 4 links and 9 total link strength. The most frequently repeated keywords of this cluster show that the studies using these words focus on the recognizing and reporting of biological assets within the framework of IAS 41 [3].

Cluster 2 (Green): There are 16 keywords in this cluster. The phrase ``fair value'' is the strongest keyword of this cluster with 20 links and 58 total link strength. ``Historical cost'' ranks second among the strongest keywords in this cluster with 9 links and 21 total link strength, and ``Farm accounting'' ranks third with 13 links and 17 total link strength. This is followed by the keyword ``Bearer plants'' with 6 links and 10 total link strength. The most frequently repeated keywords of this cluster show that the studies using these keywords focus on the valuation of biological assets [10,11].

Cluster 3 (Blue): This cluster consists of 12 keywords. Keywords with lower number of links and total link strength compared to the red and green clusters came together in this cluster. The phrase ``Dairy Farms'' appears as the strongest keyword of this cluster with 8 links and 9 total link strength. ``Economic performance'' is the second strongest keyword in this cluster with 4 connections and 5 total connection strengths. Each of the words ``Farm accounting data network'', ``Eco-efficiency'' and ``Subsidies'' ranks third with 4 links and 4 total link strength. When the most frequently repeated keywords in this cluster are examined, it is seen that the studies in which these keywords are used together focus on the theme of performance and efficiency measurement with the data obtained from “Farm Accounting Data Network” (FADN) in dairy farms [12,13].

Cluster 4 (Yellow): There are 12 keywords in this cluster. The word ``Agriculture'' is the strongest keyword in this cluster, with 18 links and 33 total link strengths. The word ``Agricultural Accounting'', which is the second strongest keyword of the yellow cluster with 10 links and 14 total link strength, is followed by the words ``Farm'' with 8 link and 9 total link strength, and ``Income'' with 5 links and 6 total link strength. When the most frequently repeated keywords in this cluster are examined, it is seen that the studies in which these keywords are used together focus on the theme of accounting practices in agricultural enterprises [[14], [15]].

Cluster 5 (Purple): 8 keywords have come together in this cluster and the word ``Technical efficiency'' is the strongest keyword in this cluster with 4 links and 10 total link strength. The second strongest keyword of this cluster with 30 link and 8 total link strength is ``Data development analysis'', followed by ``Scale efficiency'' with 2 links and 6 total link strength, and ``Profitability'' with 4 links and 4 total link strength. When the most frequently repeated keywords in this cluster are examined, it is seen that the studies in which these words are used together focus on the theme of profitability and efficiency analysis in agricultural enterprises [16].

Bibliographic coupling is the citation of a third publication by two publications. The occurrence of a match when two different scientific studies (A and B) together refer to the same study is an indication of bibliographic coupling between A and B, in other words an implicit relationship between these two studies [17].

Analysis of bibliographic coupling was performed in the context of sources and documents. When examining the bibliographic coupling analysis of sources, a threshold of 1 was set for the minimum number of documents and the minimum number of citations for a source to be included. Out of 133 sources, 91 sources met this criterion. The largest set of connected sources consists of 64, as some of the 91 sources in the network are not connected to each other. The bibliographic coupling analysis resulted in 4 clusters with 193 links and a total link strength of 893. The resulting image of the analysis is shown in Fig. 6.

In the network map, sources located close to each other indicate publications with a high bibliographic coupling link, meaning they are frequently co-cited together. The bibliographic coupling analysis resulted in four clusters, with the major clusters represented by the colors red and green. The red cluster consists of 22 sources, while the green cluster consists of 16 sources. These clusters highlight the publications that have strong bibliographic connections within their respective groups.

The journal “Custos e Agronegocio Online” in the green cluster has the highest number of links (24) and total link strength (354). This is followed by “Agricultural Economics” in the yellow cluster with 32 links and 226 total link strength. These two journals, which have the strongest bibliographic coupling, include publications in the field of agricultural economics and policies. Although the journal “Spanish Accounting Review” in the green cluster has 2 publications, it appears as second strongest resource with 18 links and 197 total link strength. The “Spanish Journal of Finance and Accounting”, which is also included in the same cluster and has 1 publication, is among the journals with strong bibliographic coupling with 21 links and 124 total link strength. The green cluster mainly consists of resources related to the field of accounting, apart from the journal ``Custos e Agronegocio Online'' which is publishing in the field of agricultural economics. For example, journals such as Spanish Accounting Review, Spanish Journal of Finance and Accounting, British Accounting Review, Review of Accounting Studies, Asia-Pacific Journal of Accounting and Economics are part of this cluster.

When examining the bibliographic coupling analysis of documents, a threshold of 1 was set for the minimum number of citations. Out of 190 documents, 129 met the criteria, forming the largest set of connected sources consisting of 92 documents, while some of the 129 documents in the network were not connected to each other.

Table 1 presents the top 10 documents with the highest bibliographic coupling links in farm accounting research.

The document with the second highest bibliographic coupling connection with 110 total link strength is Argilés-Bosch et al. [1]. Argilés-Bosch et al. [2]'s study with a total connection strength of 109 is the third study with the highest total connection strength. According to the results of the analysis, it is seen that the studies made by Spanish researchers in the first three ranks are the strongest documents in terms of bibliographic coupling.

As a result of the analysis, 5 clusters with 354 links and 863 total link strengths were obtained. Fig. 7 shows the resulting network image.

It is seen that documents located close to each other on the network map have high bibliographic coupling link. It is seen that the studies in the purple-colored cluster in the center of the network map consist of studies with high link numbers and total link strength. In Table 1 above, studies by Spanish researchers listed in the top three have strong bibliographic coupling links with documents both in the same cluster and in other clusters, especially in the green cluster.

In the co-citation analysis, in which the cited references as the analysis unit are taken into account, the minimum number of citations threshold value for a cited references are determined as 5. Of the 5046 cited references, 30 met the threshold. The largest set of connected cited references consist of 26 items as some of the 30 cited references in network are not connected to each other. Table 2 shows, the number of citations and total link strength of the first 10 articles that meet the default value.

Co-citation analysis tracks pairs of papers that are cited together in the source articles. When the same pairs of papers are co-cited by many authors, clusters of research begin to form. Co-citation analysis is a method used to determine the thematic similarity between two papers. Two papers are said to be co-cited if they both appear in the reference list of a third paper [18].

As a result of the network analysis, 2 clusters with 249 links and 563 total link strength were obtained. Fig. 8 shows the resulting network image.

As seen in Fig. 8, the references are divided into 2 clusters. The cluster in red is the largest cluster with 14 items, the total link strength of the references in this cluster is high. Among the studies with the greatest total link strength, Argilés and Slof [4]'s “New opportunities for farm accounting” published in the “European Accounting Review” is in this cluster. In this cluster, another study with the greatest link strength, Elad [5]'s study titled “Fair value accounting in the agricultural sector: some implications for international accounting harmonization” published in the “European Accounting Review”. The study in the top four ranks with the highest total link strength given in Table 8 are in the red cluster. There are 12 items in the green cluster. The top three studies of this cluster with the highest total link strength are Elad and Herbohn [6], Bohusova et al. [7] and Damian et al. [8].

## Limitations

It is important to acknowledge the limitation of using only the ISI database. It should be considered that different results can be reached when other studies in the field of agricultural accounting, which are not indexed in the ISI database, are examined. Future studies should consider exploring farm accounting research using data from different databases and employing alternative analysis methods.

## Ethics Statement

The research did not involve any human subjects or animal experiments. No data was collected from social media platforms.

## CRediT authorship contribution statement

**Mustafa Kıllı:** Conceptualization, Methodology, Data curation, Software, Formal analysis, Writing – original draft. **İlker Kefe:** Conceptualization, Methodology, Formal analysis, Visualization, Writing – review & editing.

## Data Availability

Bibliometric Network Mapping of Farm Accounting Studies Using a Comprehensive Dataset (Original data) (Mendeley Data).
